# Association of potentially inappropriate medications with outcomes of inpatient geriatric rehabilitation: A prospective cohort study

**DOI:** 10.1007/s00391-017-1328-x

**Published:** 2017-10-25

**Authors:** Madeleine Bachmann, Jan Kool, Peter Oesch, Marcel Weber, Stefan Bachmann

**Affiliations:** 1 Klinik für Rheumatologie und internistische Rehabilitation, Rehabilitation Center Kliniken Valens, Taminaplatz 1, 7317 Valens, Switzerland; 2 Medical Faculty, University of Zurich, Pestalozzistraße 3, 8091 Zürich, Switzerland; 3 Klinik für Rheumatologie, Stadtspital Triemli, Birmensdorferstraße 497, 8063 Zürich, Switzerland; 4 Department of Geriatrics, Inselspital, University of Bern Hospital, University of Bern, Freiburgstraße 10, 3010 Bern, Switzerland

**Keywords:** Rehabilitation, Quality of life, Outcome assessment, Prospective study, Mobility, Rehabilitation, Lebensqualität, Outcome-Assessment, Prospektive Studie, Mobilität

## Abstract

**Background:**

Higher age is associated with multimorbidity, which may lead to polypharmacy and potentially inappropriate medication (PIM).

**Objective:**

To evaluate whether PIM on admission for geriatric inpatient rehabilitation is associated with rehabilitation outcome regarding mobility and quality of life.

**Material and methods:**

A total of 210 patients were included. Medications at hospital admission were analyzed with the Screening Tool of Older Persons’ potentially inappropriate Prescriptions (STOPP) and the number of PIMs individual patients were taking was determined. The study population was then divided into two groups, one with and one without PIM. The main rehabilitation outcomes, quality of life and mobility, were assessed on admission and discharge. Associations between PIM and the main outcomes were analyzed using the two-tailed Student’s *t*-test and Spearman correlations.

**Results:**

In total 131 PIMs were identified by STOPP. Of the patients 91 (43%) were taking at least 1 PIM, and 119 patients (57%) were not taking any PIM. Patients with no PIM had a significantly better quality of life on admission (*p* < 0.05) and discharge (*p* < 0.005). The number of PIMs was not associated with the rehabilitation outcomes mobility and quality of life (Spearman’s ρ = −0.01, *p* = 0.89 and ρ = −0.02, *p* = 0.7, respectively). The quality of life and mobility increased identically in both groups from admission to discharge but the group with PIM did not reach the levels of those without PIM.

**Conclusion:**

The use of PIM may have a negative impact on the quality of life of elderly people but patients with and without PIM achieved comparable improvements in quality of life and mobility. Further studies are required to assess the long-term outcomes of patients taking PIM following inpatient rehabilitation.

## Zusammenfassung

**Hintergrund:**

Multimorbidität im höheren Alter kann zu Polypharmazie und potenziell nichtgeeigneter Medikation („potentially inappropriate medication“, PIM) führen.

**Ziel:**

Prüfung eines Zusammenhangs zwischen PIM bei Eintritt in die Rehabilitationsklinik und den Resultaten bezüglich Mobilität und Lebensqualität nach einer stationären geriatrischen Rehabilitation.

**Material und Methoden:**

Es wurden 210 Patienten in die Studie aufgenommen. Die Eintrittsmedikation wurde gemäß den Kriterien des Screening Tool of Older Persons’ potentially inappropriate Prescriptions (STOPP) überprüft. Die Studienteilnehmer wurden anschließend in 2 Gruppen zugeordnet, eine mit und eine ohne PIM. Die Haupt-Outcomes Mobilität und Lebensqualität wurden bei Ein- und Austritt erhoben. Zusammenhänge zwischen PIM und den Hauptresultaten wurden statistisch mit dem Student’s *t*-Test und mithilfe der Spearman-Korrelationen überprüft.

**Ergebnisse:**

Gemäß den STOPP-Kriterien wurden 131 PIM identifiziert. Es wiesen 91 Patienten (43 %) mindestens eine PIM auf, 119 Patienten (57 %) erhielten keine PIM. Patienten ohne PIM hatten eine signifikant bessere Lebensqualität bei Klinikeintritt (*p* < 0,05) und -austritt (*p* < 0,005). Die Anzahl der PIM zeigte keinen Zusammenhang mit den Rehabilitation-Outcomes Mobilität und Lebensqualität (Spearman ρ = −0,01, *p* = 0,89 respektive Spearman ρ = −0,02, *p* = 0,7). Für beide Gruppen ergab sich eine identische Verbesserung der Mobilität und der Lebensqualität von Ein- zu Austritt, aber die Patienten mit PIM erreichten nicht dieselben Mobilitäts- und Lebensqualitätslevel wie Patienten ohne PIM.

**Diskussion:**

„Potentially inappropriate medication“ kann einen negativen Effekt auf die Lebensqualität älterer Menschen haben, aber Patienten mit und ohne PIM erreichten eine vergleichbare Verbesserung der Lebensqualität und der Mobilität. Weitere Studien sind notwendig um den Langzeiteinfluss von PIM nach einer stationären Rehabilitation zu untersuchen.

## Introduction

The proportion of older people in western countries is increasing. Higher age is associated with multimorbidity, which is a widespread medical problem often leading to polypharmacy. Polypharmacy is associated with an increased risk of prescription of potentially inappropriate medications (PIM). This study investigated whether PIM have an association with rehabilitation outcomes of an inpatient geriatric rehabilitation in Switzerland. The use of PIM is often found in a population of older persons [7, 16], and may include medications that are not indicated, which are not correctly prescribed, or which are not appropriate for elderly people. In an evaluation of medication use over a period of 7 days, Fialová et al. showed that over 95% of elderly persons took at least one medication and polypharmacy was documented in 51% of the patients [7]. At least one potentially inappropriate drug was taken by 19.8% of the patients. Frequently prescribed PIMs were vasodilators, benzodiazepines, heart medication, antidepressants and anticoagulants (e. g. warfarin) and PIMs were associated with an age of 75–79 years, frailty and polypharmacy [21]. Frail people are at increased risk of adverse drug events, e. g. falls, hospitalization and mortality. Landi et al. have shown that PIM may have adverse effects, which decrease the physical performance of patients, especially in community-dwelling elderly people over 80 years of age [13]. The physical performance decreased more in patients taking two or more inappropriate drugs in comparison with non-users or patients taking only one inappropriate drug, indicating that the association between impaired physical performance and PIM becomes stronger when the number of inappropriate drugs increases. Users of PIM were more likely to have cognitive impairment in comparison with non-users, were less likely to be physically active, and had a higher number of medications.

To identify PIM most of the previous studies used the criteria of Beers [4], a list of medications that should be avoided in elderly patients due to frequent adverse drug events; however, the drugs on the Beers list are mostly administered in the USA and are not available in Europe. Alternatives in the European setting include the Priscus list [11], a list of medications administered in Germany that should be avoided, the Screening Tool to Alert doctors to the Right Treatment (START) or the Screening Tool of Older Persons’ potentially inappropriate Prescription (STOPP), which focus on avoiding inappropriate drugs (STOPP) or to identify undertreatment medications (START) [10]. Gallagher and O’Mahoney showed that the STOPP criteria identified a significantly higher proportion of patients requiring hospitalization as a result of PIM-related adverse events than Beers’ criteria [9].

Rehabilitation is known to have a beneficial effect on the physical performance of elderly patients [15]. Baztán et al. showed that patients cared for in acute geriatric rehabilitation units were more likely to live at home after discharge, and that the effects were maintained 3 months after discharge [3]. In another meta-analysis Bachmann et al. found that inpatient rehabilitation specifically designed for geriatric patients has the potential to improve outcomes regarding function (Barthel index), nursing home admissions and mortality [1]. Inpatient rehabilitation was significantly more beneficial regarding functional outcomes at discharge and in the long term. In a cohort study over a period of 12 months Bellelli et al. showed that advanced age, living alone, cognitive impairment, delirium and poor functional status at discharge from a rehabilitation unit were the main predictors of subsequent institutionalization [5]. Multimorbidity and delirium were predictors of rehospitalization. Multimorbidity, often leading to (inappropriate) polypharmacy, is furthermore a predictor for mortality and should be considered at discharge for secondary prevention programs [5]; however, it is unknown whether PIM at admission to geriatric inpatient rehabilitation has an association with rehabilitation outcome. In a recently published study mobility was the main predictor for successful return home after inpatient geriatric rehabilitation [12].

The purpose of this study was to investigate the association of PIM at admission to an inpatient rehabilitation program with the outcome of rehabilitation regarding mobility and quality of life. The hypothesis was that patients taking PIM would have a less favorable rehabilitation outcome regarding mobility and quality of life than patients not taking PIM.

## Methods

### Design

This prospective, single center, cohort study was conducted in the rehabilitation center Walenstadtberg (Kliniken Valens, Switzerland), a clinic for geriatric inpatient rehabilitation. This study was part of a study to evaluate predictive factors for living at home after geriatric rehabilitation [12]. A sample size of 120 patients was required for the analysis, with 60 additional patients to account for loss to follow-up. Patients were recruited consecutively between February and November 2014. Recruitment exceeded expectations and a final total of 210 patients were included.

### Geriatric rehabilitation

A standardized geriatric assessment, together with individual goal setting, was performed to plan rehabilitation interventions. Patients received a mean of 3 treatment sessions per day (in total 2 h per day), 6 days per week, for 3 weeks. Treatment was based on patient needs and included individual physical and occupational therapy, medical exercise training, aquatic exercise and passive modalities. The indications for any treatment, including medication, was the responsibility of the interdisciplinary rehabilitation team.

### Subjects

All patients aged 65 years or more who were referred for geriatric inpatient rehabilitation were eligible to enter the study. Inclusion criteria were sufficient understanding of the German language to answer the questionnaires and to provide written informed consent. Exclusion criteria were medical conditions that interfered with completing the study questionnaires, e. g. severe psychiatric disorders, dementia, and severe hearing and visual impairments.

### Data collection

Immediately after admission to the rehabilitation centre, the list of drugs taken by the participants was checked by a researcher (M. Bachmann), based on the referral letter or the individual medication scheme, and based on the admission diagnosis assessed according to the STOPP criteria [10] regarding appropriateness. Any doubts regarding indications for the medications were discussed and resolved in agreement with a clinician experienced in geriatric rehabilitation (S. Bachmann). The cohort was then separated into two groups: one group of patients without PIM and the other group with PIM, defined as at least one PIM.

### Assessments

In both groups baseline demographic data regarding age, sex, education, marital status, housing situation, need for social support (modified medical outcomes study social support survey; mMOS-SSS [18]) and the principal medical diagnosis according to the International Classification of Diseases and Related Health Problems (ICD-10) were recorded at admission. Furthermore, cognition (mini-mental state examination, MMSE [8]), activities of daily living (ADL and self-care index Selbstpflege-Index SPI from ePA-AC [ergebnisorientiertes PflegeAssessment AcuteCare; 2]) and vulnerability (vulnerable elders survey, VES-13 [22]) were also assessed.

Within the first 3 days after admission the quality of life (EQ-5D index, EuroQol group) [6] and comorbidities (cumulative illness rating scale; CIRS) [14] were assessed. To assess mobility, the timed up and go (TUG) test [17] was used. To enable analysis of all patients including those who were unable to stand up or walk, the results of the TUG were recoded into an ordinal scale according to the study by Kool et al. [12]. A time of up to 10 s was coded as 1, 11–20 s as 2, 21–30 s as 3, 31 s or more as 4 and unable to perform test as 5. At discharge the MMSE, SPI, EQ-5D index and TUG tests were repeated. Differences between the groups were compared regarding assessment results at admission vs. assessment results at discharge. Furthermore, associations between the independent variables, the number of PIM, and the dependent variables (changes in mobility and quality of life) were evaluated.

### Statistical analysis

Between-group comparisons of data with a normal distribution were performed with the two-tailed Student’s *t*-test. Significance level was set at *p* < 0.05. Because the number of PIMs was not normally distributed and mobility was assessed with an ordinal scale, we used Spearman’s correlations to analyze the association between the number of PIMs and the change in mobility and quality of life. Improvements in mobility and quality of life during geriatric rehabilitation depend on multiple factors; therefore, significant (*p* < 0.05) correlations >0.1 were considered relevant. Analyses were performed with SPSS version 22 (SPSS, Chicago, IL, USA).

## Results

### Recruitment and patient characteristics

**Fig. 1:**
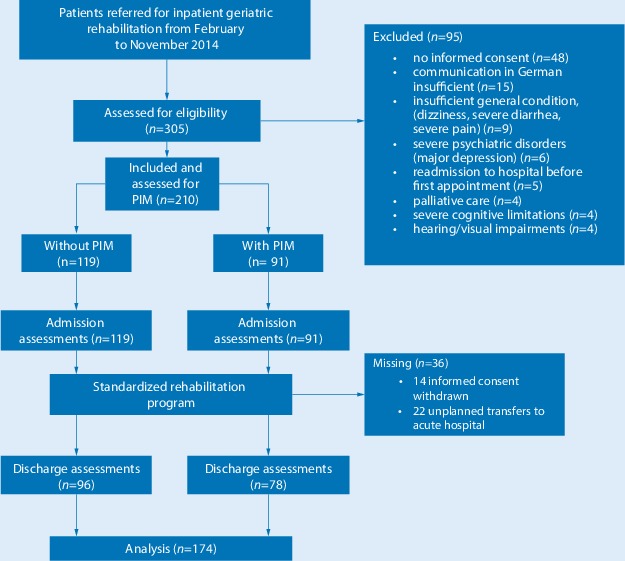
Patient recruitment, study flow and data collection. *PIM* potentially inappropriate medication

In the period from February to November 2014 a total of 305 patients were assessed for study eligibility, of whom 95 were excluded. The main reasons for exclusion were not consenting to participation (*n* = 48) and insufficient understanding of German (*n* = 15) preventing the completion of questionnaires. During the rehabilitation program 14 patients withdrew their informed consent and 22 patients underwent unplanned transfer to an acute hospital due to acute deterioration of their health status; therefore, data from 174 patients were analyzed (Fig. 1).

### Demography

Details of the baseline characteristic of both groups are presented in Table 1.

**Table 1:** Baseline characteristics of patients included in the study

	Group without PIM (*n* = 119)	Group with PIM (*n* = 91)	*p*-value (NS=not significant)
Women, *n*	68	45	NS
Men, *n*	51	46	NS
Age (SD), years	75.5 (6.94)	75.5 (10.45)	NS
*Referral from*			
Acute hospital, *n*	110	78	NS
Own home,* n*	9	13	NS
*Place of residence (prior to acute hospital stay and/or rehabilitation)*			
Urban	12.10%	13.50%	NS
Rural	87.90%	86.50%	NS
*Type of residency*			
Living alone	29.30%	12.40%	NS
Living alone with an auxiliary person	12.90%	18.50%	NS
With partner/child	42.30%	46.90%	NS
With partner/child and an auxiliary person	13.70%	17.30%	NS
Nursing home	1.80%	4.90%	NS
*Diagnosis*			
Osteoarthritis	22.68%	18.69%	NS
Cancer	16.81%	9.89%	NS
Spinal diseases	14.28%	18.69%	NS
Pulmonary diseases	13.45%	10.99%	NS
Fractures	10.92%	16.48%	NS
Other internal diseases	10.10%	12.08%	NS
Cardiovascular diseases	8.40%	10.99%	NS
Inflammatory rheumatoid diseases	3.36%	2.19%	NS
Duration of stay (SD), days	20.76 (11.25)	22.08 (8.93)	NS
mMOS–SSS (SD) (scale 19–95; 95 = best social support)	34.55 (6.65)	34.03 (7.21)	NS
MMSE (SD) (scale 0–30; >26 cognition unimpaired)	26.49 (2.76)	26.15 (3.21)	NS
CIRS (SD) (scale 0–56; 56 maximum illness load/comorbidity)	11.13 (6.02)	13.24 (6.61)	0.02
VES-13 (SD) (scale 1–10; ≥3 = vulnerable)	4.49 (2.99)	5.38 (2.80)	0.02
SPI (SD) (scale 0–40; 40 = fully independent)	37.08 (3.46)	35.77 (4.99)	0.03

*PIM* potentially inappropriate medications, *mMOS-SSS* modified medical outcomes study social support survey, *MMSE* mini-mental state examination, *CIRS* cumulative illness rating scale, co-morbidities, *VES-13* vulnerable elders survey, *SPI* self-care index (Selbstpflege-Index) and *ADL* activities of daily living, *SD* standard deviation

Of the patients 113 were male with a mean age of 76.2 years (SD 10.0 years) and 97 were female with a mean age of 74.8 years (SD 6.6 years). The main diagnosis was osteoarthritis, followed by spinal diseases, fractures and pulmonary diseases. Further diagnoses were cancer, cardiovascular diseases, other internal diseases and inflammatory rheumatoid diseases.

Analysis of all medications based on the referral letter according to the STOPP criteria revealed that 91 patients (43%) took at least 1 PIM, and 119 patients (57%) did not take any PIM. In the group taking PIM there were more males than females (68 vs. 51), while in the group who did not take any PIM there were slightly more females than males (45 vs. 46). The mean age in both groups was the same. A total of 110 patients without PIM were referred to rehabilitation after an acute hospital stay, whereas 78 patients taking PIM came from an acute hospital. Only 9 patients not taking PIM came from their own home and 13 patients in the group with PIM. Regarding type of residency prior to acute hospital or rehabilitation, more patients with PIM were living in nursing homes (4.9% vs. 1.8%).

### Potentially inappropriate medications (PIM)

In total 131 PIM were identified by using the STOPP criteria, 60 people had 1 potentially inappropriate prescription (65.93%) and 31 patients had 2 (27.47%) or more (6.59%). The highest number of PIM according to the STOPP criteria were found in the category “central nervous system and psychotropic drugs”, with 48 PIM. The main drugs in this group were benzodiazepines and neuroleptics, the category “cardiovascular system” followed with 39 PIM, mostly concerning beta-blockers and diuretics. Further details are shown in Table 2.

**Table 2:** Details of the distribution of potentially inappropriate medications (PIM) according to Screening Tool of Older Persons’ potentially inappropriate Prescription (STOPP) criteria [10]

Category	Number of PIM	Most frequently used substance recorded
Central nervous system and psychotropic drugs	48	Lorazepam, bromazepam, flupentixol+melitracen, quetiapine, pipamperone
Cardiovascular system	39	Bisoprolol, aspirin, furosemide, torasemide
Gastrointestinal system	29	Proton pump inhibitors (PPI)
Analgesic drugs	8	Oxycodone
Musculoskeletal system	5	Non-steroidal anti-inflammatory drugs (NSAID), etodolac, diclofenac, mefenamic acid, naproxen
Respiratory system	2	Prednisone, ipratropium+salbutamol
Urogenital system	0	–
Endocrine system	0	–
Drugs that adversely affect those prone to falls (>1 fall in past 3 months)	0	–
Duplicate drug classes	0	–
Total	131	–

### Rehabilitation outcomes

The CIRS score, the VES-13 score and the SPI at admission were significantly different between the groups (*p* < 0.05), showing that the group without PIM had less comorbidities, was less vulnerable and less handicapped in ADL than the group with PIM (Table 1). At discharge these differences regarding VES-13 score and SPI were unchanged (*p* < 0.05).

**Fig. 2:**
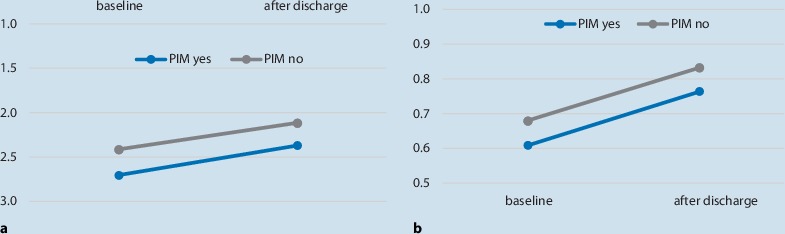
Improvement in mobility and quality of life (QOL) in the groups without potentially inappropriate medication (PIM) and with PIM at baseline and at discharge. **a** Changes in mobility in patients with and without PIMs. Y-axis: TUG 1-5 (1= best mobility, 5 unable to perform the test). **b** Changes in QOL in patients with and without PIMs. Y-axis: EQ5D-Index (1=best level of QOL)

Analysis of the EQ-5D index showed significant differences at admission (*p* < 0.05) and discharge (*p* < 0.005), indicating that the participants without PIM had a significantly better quality of life than those with PIM (Table 3). Data for the TUG also showed better results in the group without PIM; however, these differences were not significant. Changes in mobility and quality of life were comparable in patients with and without PIM (Fig. 2).

**Table 3:** Main outcomes of EQ5D and TUG tests in both groups at admission and discharge

	Without PIM Mean (SD)	With PIM Mean (SD)	*p*-value
*EQ-5D index* *(1 = best level of quality of life)*			
At admission	0.69 (0.26)	0.61 (0.28)	0.045
At discharge	0.87 (0.15)	0.78 (0.22)	0.003
*TUG* *(coded to 1–5, where 1 = best mobility and 5=unable to perform the test)*			
At admission	2.41 (1.22)	2.70 (1.24)	0.09
At discharge	2.11 (1.12)	2.37 (1.17)	0.10

*PIM* potentially inappropriate medications, *EQ-5D* EuroQol group 5 dimensions, *TUG* timed up and go test

Although there was a difference between the two groups at entry and discharge regarding mobility and quality of life, there was no association in the correlation analysis between the number of PIM and the change in mobility (Spearman’s ρ = −0.01, *p* = 0.89 and ρ = −0.02, *p* = 0.7, respectively). Nevertheless, improvements in mobility were associated with improved quality of life (Spearman‘s ρ = 0.20, *p* = 0.01).

## Discussion

To our knowledge this is the first study to evaluate the effects of PIM at admission to a geriatric inpatient rehabilitation program on rehabilitation outcomes at discharge. In our study population 43% of patients were taking at least one PIM at admission. Patients without PIM had a significantly better quality of life at admission (*p* < 0.05) and discharge (*p* < 0.005); however, during rehabilitation the quality of life and mobility of patients with and without PIM increased identically, and the number of PIM was not associated with these rehabilitation outcomes.

In the study of Mann et al. the main group of PIM in Austrian nursing homes was antipsychotic drugs, followed by benzodiazepines, non-steroidal anti-inflammatory drugs (NSAIDs) and opiates [16]. The main groups of PIMs in our study were antipsychotic drugs and benzodiazepines, followed by beta-blockers and diuretics. In the multicenter Austrian study by Mann et al. [16] with 1844 patients, the prevalence of residents taking at least one PIM was 70.3%, whereas in our population only 43.3% of the patients were taking PIM. This difference may arise from the fact that different patient populations were studied. Our patients were community-dwelling persons needing inpatient rehabilitation after an acute hospital stay because of illnesses or an operation, whereas Mann et al. assessed PIM in a nursing home setting. Besides the frequency of PIM, there were no major differences regarding the groups according to the STOPP criteria. In both settings antipsychotic drugs and benzodiazepines were the most prescribed PIM. Fialová et al. showed in an outpatient setting that older people and those living alone were more likely to be taking one or more PIM [7]. Overall, the mean age of the study population was 82.2 years, the majority of patients were women and most lived alone. Polypharmacy was documented in 51% of patients, and 19.5% of patients were taking at least one inappropriate medication. The patients included in our group were slightly younger (mean age 75.5 years) and, in contrast to other studies, more men were taking PIM. This might reflect the fact that men in Switzerland are slightly more prone to a worse health status than women [23]. We found no major differences compared with the results of the study by Gallagher et al. who first published information on the STOPP criteria [9]. The study population of Landi et al. is also comparable to ours, since they included a total of 364 patients [13] and 26% (*n* = 94) were taking at least 1 inappropriate medication. The most frequently prescribed PIM were benzodiazepines, followed by antihypertensive drugs. Both drug groups (central nervous system drugs and cardiovascular drugs) were also the most prominent PIM in our cohort. Onder et al. showed that during a hospital stay 28.6% of patients were taking inappropriate drugs, 23% took 1 inappropriate drug and 5.6% took 2 or more inappropriate drugs [19]. In this acute hospital setting, the intake of PIM had no significant effect on hospital outcomes, such as mortality, length of stay, or adverse drug reactions.

In our study population, patients who were not taking any PIM had a significantly better quality of life at admission and discharge than patients who were taking PIM. Furthermore, patients who were not taking any PIM were less impaired at admission and discharge and more independent at the end of the rehabilitation. Interestingly, the group with PIM showed a significantly higher comorbidity index at admission than the group who were not taking any PIM, perhaps indicating that a higher illness load may directly lead to an increase in medications and therefore also to taking medications that potentially are not indicated. Nevertheless, it seems that PIM do not have a negative impact on the rehabilitation course, as quality of life and mobility increased identically in both groups from admission to discharge. Patients with PIM generally had lower levels of increased quality of life and mobility than those without PIM. Landi et al. showed that community-dwelling older persons in Italy taking PIM had a significant negative association regarding physical performance [13]. Patients who were taking PIM were less likely to be physically active and showed poorer results in the short physical performance battery. In another study from Runganga et al. in a post-acute outpatient transitional care setting, which is designed to facilitate transitions from hospital to home for older people in Australia [20], polypharmacy (5–9 drugs) was observed in 46.7% of patients. The most prevalent PIM were antidepressants. Polypharmacy was associated with frailty, falls and poor functional outcomes. Finally, regarding function a study from Tosato et al. [24] showed that in geriatric care wards (geriatric evaluation and management units) a decline in physical function was more often identified in patients taking PIM. The decline in function increased with the number of PIM used. Our results are in line with all these findings; our patients with PIM also seemed to be more impaired than patients who were not taking any inappropriate drugs. Although patients with PIM had lower mobility levels at baseline and after rehabilitation, correlation analysis revealed no statistical association between PIM and mobility; however, the mobility of patients taking PIM at hospital entry did not increase to the same outcome level reached by patients who were not taking any PIM despite the fact that they underwent the same rehabilitation program; this might lead to poorer outcomes in the long term. Finally, one could argue that rather than PIM the disease itself may have altered the outcome results, since different diseases may have different disease courses and therefore also different outcomes regarding quality of life and mobility; however, in our study the main diagnoses and diseases were equally distributed, with no statistical differences at baseline. Therefore, we believe that this possible effect of the disease itself would also have appeared in the group of patients not taking PIM.

### Strengths

One of the strengths of this study is the large sample of patients (*n* = 210). The majority of patients assigned for rehabilitation were included in the study, thus making the study representative of geriatric rehabilitation in Switzerland. We had a complete list of medications taken by every patient, therefore the evaluation of PIM was good. Furthermore, as we were able to stringently follow the patients during inpatient rehabilitation there was a low drop-out rate, and therefore the comparisons of the effects on rehabilitation outcomes are valid in this group of patients.

### Limitations

The observation time was short and the results presented here cover a period of only 3–4 weeks. We do not know if the poorer but not significantly different results regarding mobility and quality of life for patients taking PIM persist after inpatient rehabilitation. Another limitation is that we had no information regarding when the PIM were first prescribed, during the preceding hospital stay or earlier. This might create bias regarding our results, as a longer period of taking PIM may have stronger negative effects, which may lead to a poorer quality of life and lower mobility, depending on the prescribed medication. In addition, if the drug was given in the preceding hospital for the first time, PIM may lead to adverse drug reactions, such as delirium, which also may alter quality of life and mobility. Finally, the results presented here may not be generalized, since approximately 90% of all our patients came to rehabilitation after an acute hospital stay. The results are best applicable to similar patients. We assume lower generalizability, e. g. to medically stable persons over 65 years of age, independently living at home, with lower comorbidity and lower PIM rates.

### Conclusion

Overall, the results indicate that inappropriate drugs should be monitored with care, as they may have a negative impact on the mobility and quality of life of elderly people. Together with the results of our study, all published evidence so far shows that inappropriate drugs are administered to a high proportion of older people, and that this may lead to lower levels of mobility and poorer quality of life.

### Further implications

Further research is needed into the long-term outcomes of inpatient rehabilitation in patients receiving PIM, e. g. in a randomized clinical trials comparing rehabilitation with and without a special focus on PIM reduction and with 1‑year follow-up after inclusion. Future research should also focus on the question as to whether outcomes in the long term are better when PIM use is corrected in the acute hospital setting. Since PIM seem to have a negative correlation with mobility and quality of life, a long-term cost analysis should be performed to determine whether use of PIM also has a negative correlation with medical and societal costs.

### Practical conclusion

- PIM are frequently found at hospital admittance for inpatient rehabilitation (43% of all patients).
- PIM are associated with lower mobility and quality of life, but patients with and without PIM achieved comparable improvements in mobility and quality of life.
- Medications for elderly people should be monitored on a regular basis to identify and correct PIM, as they may have a negative impact on mobility and quality of life.

## References

[1] Bachmann S, Finger C, Huss A (2010). Inpatient rehabilitation specifically designed for geriatric patients: systematic review and meta-analysis of randomised controlled trials. BMJ.

[2] Bartholomeyczik S, Hunstein D (2006). Standardized assessment instruments in nursing: possibilities and limits. Pflege Z.

[3] Baztan JJ, Suarez-Garcia FM, Lopez-Arrieta J (2009). Effectiveness of acute geriatric units on functional decline, living at home, and case fatality among older patients admitted to hospital for acute medical disorders: meta-analysis. BMJ.

[4] Beers MH (1997). Explicit criteria for determining potentially inappropriate medication use by the elderly. An update. Arch Intern Med.

[5] Bellelli G, Magnifico F, Trabucchi M (2008). Outcomes at 12 months in a population of elderly patients discharged from a rehabilitation unit. J Am Med Dir Assoc.

[6] Euroqol http://www.euroqol.org/home.html. Accessed: 22.10.2017

[7] Fialova D, Topinkova E, Gambassi G (2005). Potentially inappropriate medication use among elderly home care patients in Europe. JAMA.

[8] Folstein MF, Folstein SE, Mchugh PR (1975). “Mini-mental state”. A practical method for grading the cognitive state of patients for the clinician. J Psychiatr Res.

[9] Gallagher P, O‘Mahony D (2008). STOPP (Screening Tool of Older Persons’ potentially inappropriate prescriptions): application to acutely ill elderly patients and comparison with Beers’ criteria. Age Ageing.

[10] Gallagher P, Ryan C, Byrne S (2008). STOPP (Screening Tool of Older Person’s Prescriptions) and START (Screening Tool to Alert doctors to Right Treatment). Consensus validation. Int J Clin Pharmacol Ther.

[11] Holt S, Schmiedl S, Thurmann PA (2010). Potentially inappropriate medications in the elderly: the PRISCUS list. Dtsch Arztebl Int.

[12] Kool J, Oesch P, Bachmann S (2017). Predictors for living at home after geriatric inpatient rehabilitation: a prospective cohort study. J Rehabil Med.

[13] Landi F, Russo A, Liperoti R (2007). Impact of inappropriate drug use on physical performance among a frail elderly population living in the community. Eur J Clin Pharmacol.

[14] Linn BS, Linn MW, Gurel L (1968). Cumulative illness rating scale. J Am Geriatr Soc.

[15] Luk JK, Chan CF (2011). Rehabilitation outcomes of older patients at 6 months follow-up after discharged from a geriatric day hospital (GDH). Arch Gerontol Geriatr.

[16] Mann E, Haastert B, Bohmdorfer B (2013). Prevalence and associations of potentially inappropriate prescriptions in Austrian nursing home residents: secondary analysis of a cross-sectional study. Wien Klin Wochenschr.

[17] Mathias S, Nayak US, Isaacs B (1986). Balance in elderly patients: the “get-up and go” test. Arch Phys Med Rehabil.

[18] Moser A, Stuck AE, Silliman RA (2012). The eight-item modified medical outcomes study social support survey: psychometric evaluation showed excellent performance. J Clin Epidemiol.

[19] Onder G, Landi F, Liperoti R (2005). Impact of inappropriate drug use among hospitalized older adults. Eur J Clin Pharmacol.

[20] Runganga M, Peel NM, Hubbard RE (2014). Multiple medication use in older patients in post-acute transitional care: a prospective cohort study. Clin Interv Aging.

[21] Saarelainen LK, Turner JP, Shakib S (2014). Potentially inappropriate medication use in older people with cancer: prevalence and correlates. J Geriatr Oncol.

[22] Saliba D, Elliott M, Rubenstein LZ (2001). The vulnerable elders survey: a tool for identifying vulnerable older people in the community. J Am Geriatr Soc.

[23] Swiss Federal Statistical Office (2015). Gesundheit. Taschenstatistik.

[24] Tosato M, Landi F, Martone AM (2014). Potentially inappropriate drug use among hospitalised older adults: results from the CRIME study. Age Ageing.

